# Spontaneous tumor lysis syndrome in adrenal adenocarcinoma: a case report and review of the literature

**DOI:** 10.1186/s13256-022-03263-4

**Published:** 2022-02-10

**Authors:** Mahan Shafie, Alireza Teymouri, Samaneh Parsa, Ali Sadeghian, Narjes Zarei Jalalabadi

**Affiliations:** 1 School of Medicine, Tehran University of Medical Sciences, Tehran, Iran; 2 NeuroTRACT Association, Students’ Scientific Research Center, Tehran University of Medical Sciences, Tehran, Iran; 3 Department of Internal Medicine, Imam Khomeini Hospital Complex, Keshavarz Boulevard, Post code: 1419733141 Tehran, Iran; 4 Students’ Scientific Research Center, Tehran University of Medical Sciences, Tehran, Iran

**Keywords:** Spontaneous tumor lysis syndrome, Adrenal adenocarcinoma, Solid tumor

## Abstract

**Background:**

Tumor lysis syndrome is an oncologic emergency that classically occurs following cancer therapy, although spontaneous tumor lysis syndrome can also occur in malignancies, albeit rarely. Spontaneous tumor lysis syndrome has previously been reported in some hematologic malignancies, but it rarely happens in solid tumors and seems to be associated with a higher mortality rate. This is the first case of adrenal adenocarcinoma that developed spontaneous tumor lysis syndrome.

**Case presentation:**

We present a rare case of spontaneous tumor lysis syndrome occurring in a patient previously diagnosed with adrenal adenocarcinoma. The patient was a 64-year-old Persian man with abdominal pain, hypersomnia, and fatigue who was previously diagnosed with right adrenocortical carcinoma and had undergone right adrenalectomy with regional lymph nodes resection 5 months previously. On physical examination, the patient had abdominal distension and mild tenderness at the right upper quadrant. Pitting edema was detected bilaterally in the lower extremities. Initial imaging revealed multiple and large lesions suggestive of liver metastases. The laboratory data showed hyperkalemia, hyperuricemia, hyperphosphatemia, and elevated serum creatinine level indicative of spontaneous tumor lysis syndrome in the patient. Despite immediate and intensive care with antibiotics, hydration, treatment with a hypouricemic agent, and renal replacement therapy, the patient ultimately died from multiorgan failure.

**Conclusions:**

Tumor lysis syndrome in solid tumors has high mortality. Patients susceptible to spontaneous tumor lysis syndrome must receive aggressive treatment immediately, which is crucial for preventing morbidity and mortality. Spontaneous tumor lysis syndrome may be underdiagnosed, and a high degree of clinical suspicion is needed to make the diagnosis and proceed with required interventions. Therefore, clinicians should be aware of this rare phenomenon.

**Supplementary Information:**

The online version contains supplementary material available at https://doi.org/10.1186/s13256-022-03263-4.

## Background

Tumor lysis syndrome (TLS) is an oncologic emergency that results from the release of intracellular contents including potassium, phosphate, and nucleic acids, proteins, and other metabolites into the bloodstream secondary to the rapid breakdown of tumor cells. This condition may lead to acute kidney injury, cardiac arrhythmias, seizures, and ultimately death [1]. Although TLS classically occurs following cancer therapy (chemotherapy, radiotherapy, immunotherapy, or other treatments), spontaneous TLS (STLS) can occur rarely in malignancies. STLS has previously been reported in some hematologic malignancies but is thought to be extremely rare in solid tumors [2]. Moreover, it is estimated that STLS in solid tumors is associated with more mortality and has a worse prognosis than in hematologic malignancies [3]. In our case, a patient with adrenal adenocarcinoma and subsequent adrenalectomy presented with nonspecific symptoms, confusion, abdominal pain, and electrolyte impairments, highly suggestive of spontaneous tumor lysis. To the best of our knowledge, this is the first case of adrenal adenocarcinoma that developed STLS.

## Case presentation

A 64-year-old Persian man presented with abdominal pain. He was confused and affected by hypersomnia and fatigue. The patient also had retrosternal chest pain, exertional dyspnea, nausea, vomiting, and constipation. He was previously diagnosed with right adrenocortical carcinoma and had undergone right adrenalectomy with regional lymph nodes resection 5 months previously; the histopathology revealed adrenal cortical carcinoma with a score of 6 based on Weiss criteria, and immunohistochemistry was positive for synaptophysin, inhibin, and CKAE1/AE3 (Additional file 1: Fig. S1, Additional file 2: Fig. S2, Additional file 3: Fig. S3). He had a history of hypertension, and amlodipine was his only hypertensive medication. For constipation he had received lactulose, and oxycodone was prescribed for pain relief. The patient was a heavy cigarette smoker and had been using opium for more than 20 years. At admission, the patient had a blood pressure of 120/76 mmHg and a heart rate of 107 bpm. His respiratory rate was 20 breaths per minute. A body temperature of 36.7 °C and SPO_2_ of 93% were also recorded. On physical examination, the patient had abdominal distension and mild tenderness at the right upper quadrant. Pitting edema was detected bilaterally in the lower extremities. Other physical examinations were insignificant. Chest pain and dyspnea prompted echocardiography (ECG), which was unremarkable with estimated ejection fraction of 55% and no pericardial effusion. In addition to abdominal pain, nausea, and vomiting, the patient did not have defecation and gas passage for two consecutive days. To evaluate possible gastrointestinal obstruction, consultation with the surgery department and abdominopelvic x-ray were performed. Accordingly, the possibility of obstruction was ruled out. Abdominopelvic computed tomography (CT) revealed multiple and large lesions suggestive of liver metastases. In initial laboratory results, hyperkalemia (potassium 6 mg/dl), hyperuricemia (uric acid 16.4 mg/dl), hyperphosphatemia (phosphorus 6.9 mg/dl), and hypocalcemia (calcium 7.8 mg/dl), with elevated serum creatinine level (creatinine 3.8 mg/dl), were detected. White blood cell count of 26.58 × 10^9^/L, C-reactive protein (CRP) of 45 mg/L, and alkaline phosphatase (ALP) of 1144 IU/L were indicative of an inflammatory process (Table 1). In urine analysis, significant amounts of blood and protein were found. Due to these electrolyte disturbances (hyperkalemia, hyperuricemia, and hyperphosphatemia), which fulfilled Cairo–Bishop criteria, tumor lysis syndrome was considered as an etiology of acute kidney injury for this patient, and since he had not ever received chemotherapy or radiotherapy, the patient was diagnosed as having spontaneous tumor lysis syndrome (TLS) and treated accordingly. Hydration with crystalloid intravenous fluids such as normal saline at a rate of 200 ml/hour was started as initial management to expand the intracellular volume and increase renal excretion of uric acid and other metabolites. A loop diuretic was used as add-on therapy to prevent volume overload and to wash out obstructing uric acid crystals. After ensuring a sufficient level of G6PD in the patient, rasburicase was administered to convert uric acid to more soluble and easily excreted compounds. Hyperkalemia, a life-threatening abnormality in TLS, was managed with sodium polystyrene sulfonate as a potassium-lowering agent. Insulin plus hypertonic glucose was also prescribed as a temporizing measure. In addition to massive hydration and hypouricemic agents, we considered restriction of dietary phosphate intake and administration of a phosphate binder such as sevelamer carbonate, to treat hyperphosphatemia. Blood and urine cultures were drawn, and piperacillin–tazobactam was administrated to treat possible sepsis. Due to volume overload signs (i.e., pulmonary congestion and peripheral edema), severe oliguria, and blunted consciousness during hospitalization, the patient underwent renal replacement therapy with hemodialysis on three consecutive days. Despite immediate and intensive care with hydration, treatment with a hypouricemic agent, and renal replacement therapy, the patient ultimately passed away from cardiopulmonary failure.

**Table 1:** Patient’s laboratory data during hospitalization

	On admission	Day 1	Day 2	Day 3	Day 4	Day 5	Day 6	Day 7
Na (mEq/l)	136	139	133	132	131	132	137	137
K (mEq/l)	6.0	5.3	3.7	3.2	3.1	4.5	5.1	6.0
BS (mg/dl)	55	71	-	-	57	-	55	42
Urea (mg/dl)	189	243	188	196	207	202	207	182
Cr (mg/dl)	3.8	4.0	3.6	3.7	4.4	4.4	4.3	4.6
Ca (mg/dl)	7.8	7.2	6.7	6.3	7.1	7.1	7.2	7.2
P (mg/dl)	6.9	8.5	7.2	7.9	8.6	5.7	8.5	–
Mg (mg/dl)	3.1	3.5	3.0	2.3	2.6	2.7	2.8	2.6
Uric acid (mg/dl)	16.4–	4.0	7.1	2.5	2.0	1.2	–	2.7
CPK (mg/dl)	–	174	–	–	–	–	2.6	–
LDH (mg/dl)	–	8223	–	–	–	–	–	–
WBC (count)	26,580	–	–	–	23,500	25,960	–	20,800
Hb (%)	9.8	–	–	–	10.7	9.7	–	10
Platelet (count)	486,000	–	–	–	385,000	403,000	–	341,000
PT (seconds)	15.3	–	17	–	–	–	–	–
PTT (seconds)	32	–	37	–	–	–	–	–
INR	1.17	–	1.3	–	–	–	–	–
AST (mg/dl)	360	–	–	–	–	–	–	–
ALT (mg/dl)	44	–	–	–	–	–	–	–
ALP (mg/dl)	1144	–	–	–	–	–	–	–
Bilirubin total (mg/dl)	0.8	–	–	–	–	–	–	–
CRP (mg/dl)	45	–	–	–	–	–	–	–
Albumin (g/dl)	–	–	2.6	–	–	2.1	–	–

WBC, white blood cells; Hb, hemoglobin; CRP, C-reactive protein; AST, aspartate aminotransferase; ALT, alanine aminotransferase; ALP, alkaline phosphatase; LDH, lactate dehydrogenase; CPK, creatine phosphokinase; BS, blood sugar; Cr, creatinine; PT, prothrombin time; PTT, partial thromboplastin time; INR, international normalized ratio

## Discussion and conclusion

We present a fatal case of spontaneous tumor lysis as a complication of metastatic adrenal adenocarcinoma. To our knowledge, this is the first case presentation reporting STLS in adrenal adenocarcinoma. TLS is an oncologic emergency that usually occurs after initiation of treatment in hematologic malignancies. It is rarely reported in solid tumors and may occur occasionally in solid tumors with a massive burden after receiving treatment [2]. The diagnosis of TLS is based on Cairo–Bishop criteria [4], and at least two of the following metabolic abnormalities must be present: potassium ≥ 6 mmol/L or 25% increase from baseline; phosphorus ≥ 4.6 mg/dl or 25% increase from baseline; calcium ≤ 7.0 mg/dl or 25% decrease from baseline; uric acid ≥ 8.0 mg/dl or 25% increase from baseline. Spontaneous TLS (STLS) is the development of TLS in a patient with malignancy who had not received cancer-specific therapy. STLS rarely happens in solid tumors, and it has been suggested STLS is associated with a high mortality rate according to previous studies [3].

The etiology and risk factors of STLS remain unclear and controversial in solid tumors. Large bulky tumors are considered as risk factors due to insufficient blood flow. However, our patient had underwent adrenalectomy and regional lymph node dissection. In addition, based on previous case reports, multiple liver metastases, as in our case, seemed to contribute to the development of STLS in patients with solid malignancies [5]. A systemic inflammatory syndrome may occur due to necrosis in a cancerous bulk, and resultant infection in advanced metastatic disease, which is also associated with STLS development [6, 7]. Other risk factors for development of STLS in patients with solid tumors include old age, high LDH plasma level, WBC count, and past medical history of renal impairment [5, 8].

Patients with malignancy, who are at risk of developing TLS, should receive immediate and intensive treatment due to a high mortality rate associated with this condition. Cardiac monitoring and urine output measurements should be performed. Blood electrolytes, uric acid, and creatinine should be checked every 4–6 h. Hyperkalemia can lead to cardiac dysrhythmia in these patients and must be taken into consideration, and calcium gluconate should be administrated. Other potassium-lowering strategies such as glucose/insulin infusion, beta-agonist inhalation, and oral potassium-binding agents may also be used. Calcium treatment should be avoided in asymptomatic hypocalcemia due to calcium phosphate formation in the kidneys and possible renal complications. Hypouricemic agents (allopurinol and rasburicase) can be used to reduce uric acid level. Although allopurinol inhibits formation of uric acid crystals, it is not effective in the treatment of acute TLS. On the other hand, rasburicase, with a dosage of 0.2 mg/kg/day, reduces the uric acid level quickly. Despite all these supportive and pharmacological treatment, some patients with the following indications require hemodialysis: severe oliguria or anuria, persistent hyperkalemia, hyperphosphatemia-induced symptomatic hypocalcemia, and calcium phosphate product ≥ 70 mg/dl [9]. TLS management comprises immediate initiation of supportive care and identification of high-risk patients. Aggressive IV hydration and the diuresis of the patient are of primary importance. Monitoring and treatment of any disturbance of electrolytes is considered to be vital in this condition.

Our literature review revealed 63 cases with solid tumor reporting development of STLS (Additional file 4: Table S1). The mean age of cases at time of developing STLS was 53 years, and 34 of these cases were male. This literature review showed that various tumors may develop STLS; however, the most commonly reported cancers associated with STSL were lung, colorectal, and melanoma. Almost all of them had metastases that involved different sites. Liver metastases were reported in 41 out of 63 cases (65%). Also, 18 (29%) cases were reported to have lung involvement. As discussed in previous reports, it seems that metastasis, particularly liver metastasis, may predispose patients to develop STLS. Impaired uric acid metabolism due to hepatic dysfunction following the metastases and spreading of the tumor may be an explanation for these findings [10]. The most common initial symptoms of abdominal pain, discomfort, and distention were reported in about 55% of cases. The mortality rate of these cases was 69% (44 cases died out of 63 total cases), which seems to be higher than in hematological malignancies. These data confirm the theory that STLS has worse prognosis in solid tumors than hematological malignancies. Hence, due to the poor prognosis of TLS in malignancies, particularly STLS in solid tumors, it is essential to keep in mind that TLS can occur spontaneously, and it is important to initiate immediate treatment when any suspicious symptoms are noticed.

In conclusion, TLS in solid tumors has a high mortality. Patients susceptible to STLS must receive aggressive treatment immediately, which is crucial for preventing morbidity and mortality. STLS may be underdiagnosed, and a high degree of clinical suspicion is needed to make the diagnosis and proceed with required interventions. Therefore, clinicians should be aware of this rare phenomenon.

## Supplementary Information

**Additional file 1: Figure S1.** T1-weighted MRI showing a metastasis of the adrenocortical carcinoma.

**Additional file 2: Figure S2.** T1-weighted MRI showing right adrenocortical carcinoma tumor.

**Additional file 3: Figure S3.** T1-weighted MRI showing a metastasis of the adrenocortical carcinoma.

**Additional file 4: Table S1.** Published cases of spontaneous tumor lysis syndrome in solid tumors.

## Data Availability

Data sharing is not applicable to this article as no datasets were generated or analyzed during the current study. Laboratory data and literature review are included in this published article as supplementary files.

## Abbreviations

TLS: Tumor lysis syndrome
STLS: Spontaneous tumor lysis syndrome
