# Supplementary figures and images for: Spontaneous tumor lysis syndrome in adrenal adenocarcinoma: a case report and review of the literature
Source: J Med Case Rep. 2022 Feb 10;16:52. doi: 10.1186/s13256-022-03263-4 (PMC8830132; doi:10.1186/s13256-022-03263-4)

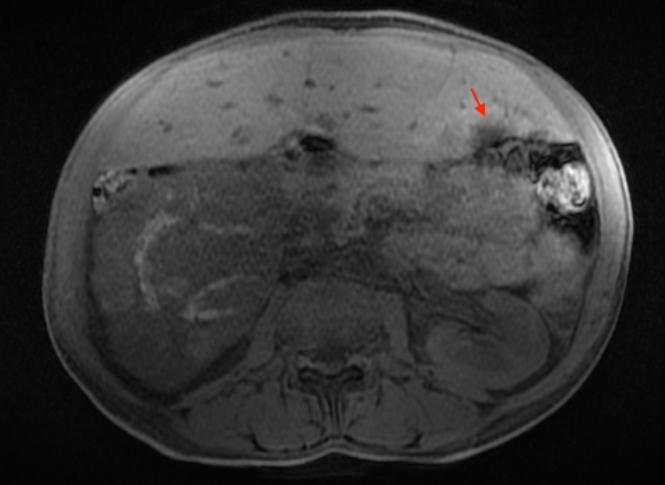

Supplement: Supplementary file 1 — Additional file 1: Figure S1. T1-weighted MRI showing a metastasis of the adrenocortical carcinoma. [file 13256_2022_3263_MOESM1_ESM.jpg]

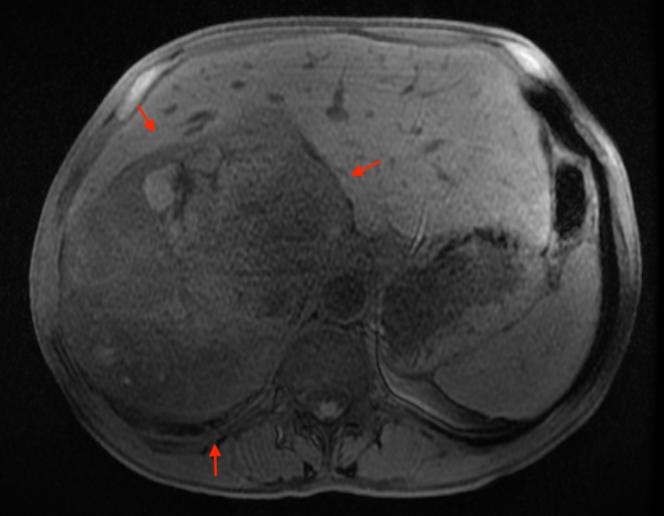

Supplement: Supplementary file 2 — Additional file 2: Figure S2. T1-weighted MRI showing right adrenocortical carcinoma tumor. [file 13256_2022_3263_MOESM2_ESM.jpg]

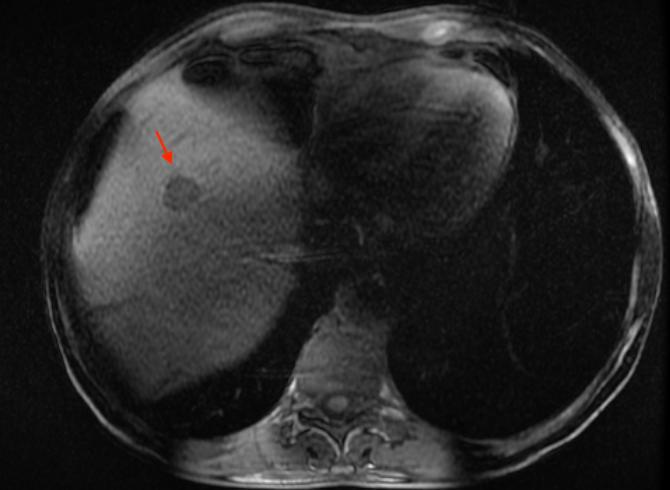

Supplement: Supplementary file 3 — Additional file 3: Figure S3. T1-weighted MRI showing a metastasis of the adrenocortical carcinoma. [file 13256_2022_3263_MOESM3_ESM.jpg]
