# Supplementary material for: Spontaneous tumor lysis syndrome in adrenal adenocarcinoma: a case report and review of the literature
Source: J Med Case Rep. 2022 Feb 10;16:52. doi: 10.1186/s13256-022-03263-4 (PMC8830132; doi:10.1186/s13256-022-03263-4)
Supplement: Supplementary file 4 — Additional file 4: Table S1. Published cases of spontaneous tumor lysis syndrome in solid tumors. [file 13256_2022_3263_MOESM4_ESM.docx]

| Table 2. Published cases of spontaneous tumor lysis syndrome in solid tumors | | | | | | | |
| --- | --- | --- | --- | --- | --- | --- | --- |
| No. | Tumor type | Age | Sex | Metastasis | Initial Symptoms | Outcome | Author/Reference |
| 1 | Neuroblastoma | 27 | Female | Lung + Liver | Abdominal Distention + Back Pain | Died | ([1](#_ENREF_1)) |
| 2 | Cholangiocarcinoma | 59 | Male | Lung + Liver | Weakness + Fatigue | Died | ([2](#_ENREF_2)) |
| 3 | Lung Small Cell Lung Cancer | 59 | Male | Liver + Mediastinal LAP | Oliguria | Died | ([3](#_ENREF_3)) |
| 4 | Caecum Adenocarcinoma | 47 | Female | Lung + Liver + Ovaries + Peritoneal Carcinomatosis | Abdominal Distention + Lower Extremity Edema + Dyspnea | Died | ([4](#_ENREF_4)) |
| 5 | Endometrial Adenocarcinoma | 59 | Female | Retroperitoneal LAP | Abdominal Pain + Somnolence | Died | ([5](#_ENREF_5)) |
| 6 | Breast Invasive Ductal Cell Carcinoma | 36 | Female | Liver + Vertebra + Left Pectoralis Minor Muscle + Left Supraventricular, Hilar, Axillary, and Retroperitoneal LAP | Chest Pain + cough + Dyspnea | Survived | ([6](#_ENREF_6)) |
| 7 | Colon Adenocarcinoma | 66 | Female | + Mediastinal, Abdominal, and Retroperitoneal LAP | Weakness + Dyspnea | Died | ([7](#_ENREF_7)) |
| 8 | Small Cell Carcinoma from Unknown Origin | 66 | Female | Liver + Peritoneal Carcinomatosis + Mediastinal LAP | Abdominal Pain | Died | ([8](#_ENREF_8)) |
| 9 | Prostate Adenocarcinoma | 49 | Male | Bone | Arthralgia + Myalgia | Survived | ([9](#_ENREF_9)) |
| 10 | Gastric Adenocarcinoma | 79 | Male | Liver + Gastrohepatic LAP | Abdominal Pain + Vomiting | Died | ([10](#_ENREF_10)) |
| 11 | Lung Small Cell Lung Cancer | 70 | Male | Liver | Back Spasms | Died | ([11](#_ENREF_11)) |
| 12 | Colorectal Adenocarcinoma | 47 | Female | Liver + Abdominal and Pelvic LAP | Abdominal Pain | Died | ([12](#_ENREF_12)) |
| 13 | Lung Small Cell Lung Cancer | 76 | Female | Liver | Lower Extremity Edema + Dyspnea | Died | ([13](#_ENREF_13)) |
| 14 | Rhabdomyosarcoma | 36 Weeks Neonate | Female | - | - | Died | ([14](#_ENREF_14)) |
| 15 | Melanoma | 59 | Male | Liver + Spleen + Adrenal + Inguinal and Pelvic LAP | Abdominal Pain + Nausea | Died | ([15](#_ENREF_15)) |
| 16 | Prostate Adenocarcinoma | 56 | Male | Liver + Spine + Pelvic Bone + Retroperitoneal, Supraclavicular, and Pelvic LAP | Abdominal Pain | Died | ([16](#_ENREF_16)) |
| 17 | Colon Adenocarcinoma | 49 | Female | Liver | Abdominal Pain + Vomiting | Died | ([17](#_ENREF_17)) |
| 18 | Ovarian Epithelial Adenocarcinoma | 49 | Female | Pleura + Peritoneal Carcinomatosis | Abdominal Pain + Vomiting | Survived | ([18](#_ENREF_18)) |
| 19 | Endometrial Adenocarcinoma | 33 | Female | Ovaries + Peritoneal Carcinomatosis | Abdominal Pain + Vomiting | Survived | ([19](#_ENREF_19)) |
| 20 | Leiomyosarcoma | 58 | Female | Lung + Peritoneal Carcinomatosis +  Mediastinal LAP | Abdominal Distention + Constipation Nausea + Dyspnea | Died | ([20](#_ENREF_20)) |
| 21 | Endometrial Adenocarcinoma | 59 | Female | Lung + Liver + Greater Omentum | Fever + Malaise | Died | ([21](#_ENREF_21)) |
| 22 | Colon Adenocarcinoma | 48 | Male | Liver | Abdominal Pain + Nausea + Anorexia + Chills | Died | ([22](#_ENREF_22)) |
| 23 | Prostate Adenocarcinoma | 69 | Male | Liver + Spine | Abdominal Pain + Vomiting + Weakness + Dizziness | Died | ([23](#_ENREF_23)) |
| 24 | Lung Small Cell Lung Cancer | 55 | Female | Liver + Mediastinal, Left Hilar, Axillary, and Supraclavicular LAP | Dyspnea | Survived | ([24](#_ENREF_24)) |
| 25 | Cervical SCC | 35 | Female | Lung + Pleura | Abdominal Pain | Died | ([25](#_ENREF_25)) |
| 26 | Lung Small Cell Lung Cancer | 53 | Male | Mediastinal and Hilar LAP | Lower Extremity Edema | Sirvived | ([26](#_ENREF_26)) |
| 27 | Osteosarcoma | 65 | Female | Lung | Abdominal Pain | Survived | ([27](#_ENREF_27)) |
| 28 | Pancreatic Adenocarcinoma | 68 | Female | Lung + Liver | Vomiting + Oliguria | Died | ([28](#_ENREF_28)) |
| 29 | Melanoma | 46 | Male | Liver + Spleen + Adrenal + Spine + Skull | Abdominal Pain + Facial Drooping | Died | ([29](#_ENREF_29)) |
| 30 | Hepatocellular Carcinoma | 26 | Female | Lung + LAP | Abdominal Pain and Distention + Tetany | Died | ([30](#_ENREF_30)) |
| 31 | Melanoma | 62 | Male | Lung + Liver + Vertebra | Back Pain | Died | ([31](#_ENREF_31)) |
| 32 | Renal Cell Carcinoma | 88 | Female | Liver + Bone | Vomiting | Died | ([32](#_ENREF_32)) |
| 33 | Testicular Cancer | 47 | Male | - | Lower Extremity Edema + Dyspnea | Died | ([33](#_ENREF_33)) |
| 34 | Lung Small Cell Lung Cancer | 65 | Male | Liver + Vertebra + Supraclavicular, Mediastinal, Hilar, Retroperitoneal LAP | Abdominal Pain and Distention | Survived | ([34](#_ENREF_34)) |
| 35 | Uterine Adenocarcinoma | 62 | Female | - | Abdominal Distention | Survived | ([35](#_ENREF_35)) |
| 36 | Pancreatic Adenocarcinoma | 56 | Female | Liver | Fatigue | Died | ([36](#_ENREF_36)) |
| 37 | GI or Ovarian Adenocarcinoma | 71 | Female | Lung + Liver + Renal + Adrenal + Skin | Skin Nodules | Died | ([37](#_ENREF_37)) |
| 38 | Colon Mucinous Adenocarcinoma | 27 | Male | Lung + Liver + Pleura + LAP | Abdominal Pain | Died | ([38](#_ENREF_38)) |
| 39 | Renal Cell Carcinoma | 56 | Male | Lung + Liver + Bone | Back Pain | Died | ([39](#_ENREF_39)) |
| 40 | Spindle Cell Sarcoma | 49 | Female | Lung + Liver + Retroperitoneal Mass | Abdominal Pain | Died | ([40](#_ENREF_40)) |
| 41 | Gastric Adenocarcinoma | 51 | Male | Liver + Bone + Adrenal + LAP | Weakness | Survived | ([41](#_ENREF_41)) |
| 42 | Cholangoicarcinoma | 66 | Male | Liver | Abdominal Pain | Survived | ([42](#_ENREF_42)) |
| 43 | Hepatocellular Carcinoma | 70 | Male | LAP | Unresponsiveness | Died | ([43](#_ENREF_43)) |
| 44 | Melanoma | 69 | Male | Liver | Rectal Bleeding | Died | ([44](#_ENREF_44)) |
| 45 | Adenocarcinoma Unknown Origin | 59 | Female | Liver + Retroperitoneal LAP | Vomiting | Died | ([45](#_ENREF_45)) |
| 46 | Hepatocellular Carcinoma | 76 | Male |  | Vomiting | Died | ([46](#_ENREF_46)) |
| 47 | Melanoma | 46 | Male | Groin and Mammary LAP | Abdominal Pain + Vomiting | Died | ([47](#_ENREF_47)) |
| 48 | Germinal Cell Tumor | 13 | Female | Peritoneum | Abdominal Distention | Survived | ([48](#_ENREF_48)) |
| 49 | Germinal Cell Tumor (Choriocarcinoma) | 22 | Male | Lung + Liver | Abdominal Distention | Died | ([49](#_ENREF_49)) |
| 50 | Hepatobastoma | 7 months | Male | - | Abdominal Distention + Vomiting | Survived | ([50](#_ENREF_50)) |
| 51 | Maxillary Sinus SCC | 53 | Male | Liver | Abdominal Pain | Died | ([51](#_ENREF_51)) |
| 52 | Lung SCC | 74 | Male | - | Anuria | Survived | ([52](#_ENREF_52)) |
| 53 | Prostate Adenocarcinoma | 72 | Male | Liver + Bone | Anorexia | Died | ([53](#_ENREF_53)) |
| 54 | Colon Adenocarcinoma | 82 | Female | Liver | Weakness | Survived | ([54](#_ENREF_54)) |
| 55 | Pheochromocytoma | 80 | Male | - | Abdominal Pain | Survived | ([54](#_ENREF_54)) |
| 56 | Hepatocellular Carcinoma | 72 | Male | - | Weakness | Died | ([54](#_ENREF_54)) |
| 57 | Germinal Cell Tumor (Endodermal Sinus) | 52 | Male | Lung + Liver + LAP | Abdominal Pain | Survived | ([55](#_ENREF_55)) |
| 58 | Germinal Cell Tumor (Seminoma) | 24 | Male | Liver + Retroperitoneal Mass | Abdominal Pain | Survived | ([55](#_ENREF_55)) |
| 59 | Gastric Adenocarcinoma | 36 | Male | Liver + LAP | Abdominal Distention | Died | ([56](#_ENREF_56)) |
| 60 | Lung Adenocarcinoma | 72 | Male | Liver | Abdominal Distention | Died | ([57](#_ENREF_57)) |
| 61 | Sacrococcygeal Teratoma | 27 Weeks Neonate | Female | - |  | Died | ([58](#_ENREF_58)) |
| 62 | Inflammatory Breast Cancer | 62 | Female | Lung + Liver + Bone Marrow | Abdominal Distention + Bone Pain | Survived | ([59](#_ENREF_59)) |
| 63 | Adenocarcinoma Unknown Origin | 50 | Male | Liver + Bone + LAP | Abdominal Distention | Died | ([60](#_ENREF_60)) |

1. Vieceli T, Tavares ALJ, de Moraes RP, Faulhaber GAM. Metastatic adult neuroblastoma with spontaneous tumor lysis syndrome: Autops Case Rep. 2020 Sep 2;10(4):e2020181. doi: 10.4322/acr.2020.181.

2. Dong J, Cao T, Tanner N, Kundranda M. When the Tumor Lyses: A Case Report on Spontaneous Tumor Lysis Syndrome: Case Rep Oncol. 2020 Aug 14;13(2):979-984. doi: 10.1159/000508947. eCollection 2020 May-Aug.

3. Alan AM, Alan O. A case of spontaneous tumor lysis syndrome in extensive-stage small-cell lung cancer: A rare oncologic emergency: Turk J Emerg Med. 2020 Jul 18;20(3):142-145. doi: 10.4103/2452-2473.290061. eCollection 2020 Jul-Sep.

4. Shaforostova I, Fiedler R, Zander M, Pflumm J, März WJ. Fatal Spontaneous Tumor Lysis Syndrome in a Patient with Metastatic Colon Cancer: A Clinical Case of Rare Oncological Emergency: Case Rep Gastroenterol. 2020 May 7;14(2):255-260. doi: 10.1159/000507648. eCollection 2020 May-Aug.

5. Chango Azanza JJ, Mathew Thomas V, Calle Sarmiento PM, Singh M, Alexander SA. Spontaneous Tumor Lysis Syndrome Due to Endometrial Carcinoma: Cureus. 2020 Mar 9;12(3):e7220. doi: 10.7759/cureus.7220.

6. Parsi M, Rai M, Clay C. You Can't Always Blame the Chemo: A Rare Case of Spontaneous Tumor Lysis Syndrome in a Patient with Invasive Ductal Cell Carcinoma of the Breast: Cureus. 2019 Nov 18;11(11):e6186. doi: 10.7759/cureus.6186.

7. Kalmbach KE, Rahmat LT, Wos JA, Daniel NJ. A Rare Oncologic Emergency: Spontaneous Tumor Lysis Syndrome in Metastatic Colon Adenocarcinoma: Clin Pract Cases Emerg Med. 2019 Oct 14;3(4):398-400. doi: 10.5811/cpcem.2019.9.43770. eCollection 2019 Nov.

8. Myint PT, Butt HW, Alrifai T, Marin C. Spontaneous Tumor Lysis Syndrome Secondary to Small-Cell Neuroendocrine Carcinoma of Unknown Origin: A Rare Case Report and Literature Review: Case Rep Oncol Med. 2019 Apr 1;2019:6375693. doi: 10.1155/2019/6375693. eCollection 2019.

9. McGhee-Jez A, Batra V, Sunder T, Rizk S. Spontaneous Tumor Lysis Syndrome as Presenting Sign of Metastatic Prostate Cancer: Cureus. 2018 Dec 8;10(12):e3706. doi: 10.7759/cureus.3706.

10. Salmón-González Z, Vieitez-Santiago M, Martino-González M, Hernández JL, Alonso-Gutierrez J. Spontaneous tumor lysis syndrome occurring in untreated gastric adenocarcinoma. Qjm. 2019;112(1):39-40.

11. Dhakal P, Rai MP, Thrasher M, Sharma M. Spontaneous tumour lysis syndrome in small cell lung cancer: a rare phenomenon. BMJ Case Rep. 2018;13(10):2018-224512.

12. Kearney MR, Chen EY, Stenzel P, Corless CL, Deloughery TG, Zivney M, et al. Colorectal Cancer-Associated Spontaneous Tumor Lysis Syndrome: a Case Report and Review of the Current Literature. J Gastrointest Cancer. 2019;50(3):668-73.

13. Dean RK, Subedi R, Lee M. Spontaneous tumor lysis syndrome in small cell lung cancer: Proc (Bayl Univ Med Cent). 2017 Dec 8;31(1):79-80. doi: 10.1080/08998280.2017.1391042. eCollection 2018 Jan.

14. Ponmudi NJ, Beryl S, Santhanam S, Beck M. Tumour lysis in newborn: spontaneous or secondary to antenatal steroids? BMJ Case Rep. 2018;4(10):2017-223107.

15. Durham CG, Herrington J, Seago S, Williams C, Holguin MH. From skin to spontaneous lysis: A case of spontaneous tumor lysis syndrome in metastatic melanoma. J Oncol Pharm Pract. 2018;24(3):221-5.

16. Serling-Boyd N, Quandt Z, Allaudeen N. Spontaneous tumor lysis syndrome in a patient with metastatic prostate cancer. Mol Clin Oncol. 2017;6(4):589-92.

17. Sommerhalder D, Takalkar AM, Shackelford R, Peddi P. Spontaneous tumor lysis syndrome in colon cancer: a case report and literature review: Clin Case Rep. 2017 Nov 13;5(12):2121-2126. doi: 10.1002/ccr3.1269. eCollection 2017 Dec.

18. Shukla DK, Gupta D, Aggarwal A, Kumar D. A Case Report of Newly Diagnosed Epithelial Ovarian Carcinoma Presenting with Spontaneous Tumor Lysis Syndrome and Its Successful Management with Rasburicase: Indian J Med Paediatr Oncol. 2017 Jul-Sep;38(3):360-362. doi: 10.4103/ijmpo.ijmpo_23_16.

19. Berger R, Waler N, Schlumbrecht M, Huang M. Spontaneous tumor lysis syndrome occurring in untreated uterine cancer: Gynecol Oncol Rep. 2017 Sep 23;22:40-42. doi: 10.1016/j.gore.2017.09.008. eCollection 2017 Nov.

20. Alaigh V, Datta D. Spontaneous Tumor Lysis Syndrome due to Uterine Leiomyosarcoma with Lung Metastases: Case Rep Crit Care. 2017;2017:4141287. doi: 10.1155/2017/4141287. Epub 2017 Sep 11.

21. Harada S, Nagaharu K, Baba Y, Murata T, Mizuno T, Kawakami K. Spontaneous Tumor Lysis Syndrome in a Patient with a Dedifferentiated Endometrial Adenocarcinoma: Case Rep Oncol Med. 2017;2017:5103145. doi: 10.1155/2017/5103145. Epub 2017 Aug 27.

22. Berringer R. Spontaneous tumor lysis syndrome in a patient with newly diagnosed metastatic colonic adenocarcinoma. Cjem. 2018;20(S2):S41-S3.

23. Ignaszewski M, Kohlitz P. Treatment-naïve spontaneous tumor lysis syndrome in metastatic prostate adenocarcinoma: An unusual suspect. Am J Emerg Med. 2017;35(9):26.

24. Boonpheng B, Murtaza G, Ginn D. Spontaneous Tumor Lysis Syndrome in a Patient with Metastatic Small Cell Lung Cancer: A Case Report: Case Rep Oncol. 2017 Apr 27;10(1):392-395. doi: 10.1159/000474937. eCollection 2017 Jan-Apr.

25. Kim YK, Ham JY, Lee WK, Song KE. Spontaneous tumour lysis syndrome in cervical cancer. J Obstet Gynaecol. 2017;37(5):679-80.

26. Kanchustambham V, Saladi S, Patolia S, Stoeckel D. Spontaneous Tumor Lysis Syndrome in Small Cell Lung Cancer: Cureus. 2017 Feb 8;9(2):e1017. doi: 10.7759/cureus.1017.

27. Catania VE, Vecchio M, Malaguarnera M, Madeddu R, Malaguarnera G, Latteri S. Tumor lysis syndrome in an extraskeletal osteosarcoma: a case report and review of the literature. J Med Case Rep. 2017;11(1):017-1241.

28. Umar J, Kalakonda A, Panebianco L, Kaur G, John S. Severe Case of Tumor Lysis Syndrome Presenting Spontaneously in a Metastatic Pancreatic Adenocarcinoma Patient: Pancreas. 2017 Apr;46(4):e31-e32. doi: 10.1097/MPA.0000000000000797.

29. Meeks MW, Hammami MB, Robbins KJ, Cheng KL, Lionberger JM. Tumor lysis syndrome and metastatic melanoma: Med Oncol. 2016 Dec;33(12):134. doi: 10.1007/s12032-016-0854-5. Epub 2016 Nov 2.

30. Agarwala R, Batta A, Suryadevera V, Kumar V, Sharma V, Rana SS. Spontaneous tumour lysis syndrome in hepatocellular carcinoma presenting with hypocalcemic tetany: An unusual case and systematic literature review. Clin Res Hepatol Gastroenterol. 2017;41(3):e29-e31.

31. Takeuchi N, Miyazawa S, Ohno Z, Yoshida S, Tsukamoto T, Fujiwara M. A Case of Spontaneous Tumor Lysis Syndrome in Malignant Melanoma: World J Oncol. 2016 Jun;7(2-3):40-44. doi: 10.14740/wjon970w. Epub 2016 Jul 1.

32. Gbaguidi X, Goodrich L, Roca F, Suel P, Chassagne P. Bulky Solid Tumors in Elderly Adults: Beware of Spontaneous Tumor Lysis Syndrome: J Am Geriatr Soc. 2016 Jan;64(1):235-7. doi: 10.1111/jgs.13901.

33. Chow M, Yuwono A, Tan R. Tumour lysis syndrome: A rare acute presentation of locally advanced testicular cancer - Case report and review of literature: Asian J Urol. 2016 Jan;3(1):49-52. doi: 10.1016/j.ajur.2015.09.005. Epub 2015 Oct 24.

34. Weerasinghe C, Zaarour M, Arnaout S, Garcia G, Dhar M. Spontaneous Tumor Lysis Syndrome in Small-Cell Lung Cancer: A Rare Complication: World J Oncol. 2015 Oct;6(5):464-471. doi: 10.14740/wjon946w. Epub 2015 Oct 26.

35. Okamoto K, Kinoshita T, Shimizu M, Okura I, Kawada A, Mizobuchi K, et al. A Case of Spontaneous Tumor Lysis Syndrome in a Patient with Ovarian Cancer. Case Rep Obstet Gynecol. 2015;461870(10):16.

36. Saleh RR, Rodrigues J, Lee TC. A tumour lysis syndrome in a chemotherapy naïve patient with metastatic pancreatic adenocarcinoma. BMJ Case Rep. 2015;29(10):2014-207748.

37. Wang Y, Yuan C, Liu X. Cutaneous metastatic adenocarcinoma complicated by spontaneous tumor lysis syndrome: A case report. Oncol Lett. 2014;8(2):905-7.

38. Frestad D, Perner A, Pedersen UG. Acute onset and rapid progression of multiple organ failure in a young adult with undiagnosed disseminated colonic adenocarcinoma. BMJ Case Rep. 2014;24(10):2014-205002.

39. Norberg SM, Oros M, Birkenbach M, Bilusic M. Spontaneous tumor lysis syndrome in renal cell carcinoma: a case report. Clin Genitourin Cancer. 2014;12(5):10.

40. Zakharia Y, Mansour J, Vasireddi S, Zakharia K, Fatakhov E, Koch C, et al. Tumor Lysis Syndrome in a Retroperitoneal Sarcoma. J Investig Med High Impact Case Rep. 2014;2(3):Jul-Sep.

41. Goyal H, Sawhney H, Bekara S, Singla U. Spontaneous acute tumour lysis syndrome in gastric adenocarcinoma: a case report and literature review. J Gastrointest Cancer. 2014;1:208-11.

42. Ali AM, Barbaryan A, Zdunek T, Khan M, Voore P, Mirrakhimov AE. Spontaneous tumor lysis syndrome in a patient with cholangiocarcinoma: J Gastrointest Oncol. 2014 Apr;5(2):E46-9. doi: 10.3978/j.issn.2078-6891.2014.012.

43. Mehrzad R, Saito H, Krahn Z, Feinstein A. Spontaneous tumor lysis syndrome in a patient with metastatic hepatocellular carcinoma. Med Princ Pract. 2014;23(6):574-6.

44. Mouallem M, Zemer-Wassercug N, Kugler E, Sahar N, Shapira-Frommer R, Schiby G. Tumor lysis syndrome and malignant melanoma. Med Oncol. 2013;30(3):012-0364.

45. Saini N, Pyo Lee K, Jha S, Patel S, Bonthu N, Kansagra A, et al. Hyperuricemic renal failure in nonhematologic solid tumors: a case report and review of the literature: Case Rep Med. 2012;2012:314056. doi: 10.1155/2012/314056. Epub 2012 May 27.

46. Kekre N, Djordjevic B, Touchie C. Spontaneous tumour lysis syndrome. Cmaj. 2012;184(8):913-6.

47. Song M, Chan CCW, Stoeckel DA. Spontaneous Tumor Lysis Syndrome in Metastatic Melanoma: World J Oncol. 2011 Aug;2(4):204-207. doi: 10.4021/wjon347w. Epub 2011 Aug 24.

48. Murray MJ, Metayer LE, Mallucci CL, Hale JP, Nicholson JC, Kirollos RW, et al. Intra-abdominal metastasis of an intracranial germinoma via ventriculo-peritoneal shunt in a 13-year-old female. Br J Neurosurg. 2011;25(6):747-9.

49. D'Alessandro V, Greco A, Clemente C, Sperandeo M, De Cata A, Di Micco C, et al. Severe spontaneous acute tumor lysis syndrome and hypoglycemia in patient with germ cell tumor. Tumori. 2010;96(6):1040-3.

50. Bercovitz RS, Greffe BS, Hunger SP. Acute tumor lysis syndrome in a 7-month-old with hepatoblastoma. Curr Opin Pediatr. 2010;22(1):113-6.

51. Abboud M, Shamseddine A. Maxillary Sinus Squamous Cell Carcinoma Presenting with Fatal Tumor Lysis Syndrome: A Case Report and Review of the Literature: Case Rep Oncol. 2009 Nov 25;2(3):229-233. doi: 10.1159/000260525.

52. Shenoy C. Acute spontaneous tumor lysis syndrome in a patient with squamous cell carcinoma of the lung: QJM. 2009 Jan;102(1):71-3. doi: 10.1093/qjmed/hcn129. Epub 2008 Oct 1.

53. Lin CJ, Hsieh RK, Lim KH, Chen HH, Cheng YC, Wu CJ. Fatal spontaneous tumor lysis syndrome in a patient with metastatic, androgen-independent prostate cancer: South Med J. 2007 Sep;100(9):916-7. doi: 10.1097/SMJ.0b013e318137a665.

54. Vaisban E, Braester A, Mosenzon O, Kolin M, Horn Y. Spontaneous tumor lysis syndrome in solid tumors: really a rare condition? Am J Med Sci. 2003;325(1):38-40.

55. Pentheroudakis G, O'Neill VJ, Vasey P, Kaye SB. Spontaneous acute tumour lysis syndrome in patients with metastatic germ cell tumours. Report of two cases. Support Care Cancer. 2001;9(7):554-7.

56. Woo IS, Kim JS, Park MJ, Lee MS, Cheon RW, Chang HM, et al. Spontaneous acute tumor lysis syndrome with advanced gastric cancer. J Korean Med Sci. 2001;16(1):115-8.

57. Feld J, Mehta H, Burkes RL. Acute spontaneous tumor lysis syndrome in adenocarcinoma of the lung: a case report. Am J Clin Oncol. 2000;23(5):491-3.

58. Jona JZ. Progressive tumor necrosis and lethal hyperkalemia in a neonate with sacrococcygeal teratoma (SCT). J Perinatol. 1999;19(7):538-40.

59. Sklarin NT, Markham M. Spontaneous recurrent tumor lysis syndrome in breast cancer. Am J Clin Oncol. 1995;18(1):71-3.

60. Crittenden DR, Ackerman GL. Hyperuricemic acute renal failure in disseminated carcinoma. Arch Intern Med. 1977;137(1):97-9.
